# The Metabolism and Transplacental Transfer of Oseltamivir in the Ex Vivo Human Model

**DOI:** 10.1155/2008/927574

**Published:** 2008-06-04

**Authors:** Kevin C. Worley, Scott W. Roberts, Roger E. Bawdon

**Affiliations:** Department of Obstetrics and Gynecology, University of Texas Southwestern Medical Center Dallas, TX 75390-9032, USA

## Abstract

Oseltamivir phosphate is extensively metabolized in the ex vivo human placenta model, and the transplacental passage of the metabolite oseltamivir carboxylate is incomplete. *Objective*. To evaluate the metabolism and transplacental transfer of oseltamivir (Tamiflu) in the ex vivo human placental model. *Study Design*. Perfusion studies were performed in six placentas from term, uncomplicated deliveries. Concentrations of oseltamivir phosphate (OP) that were 5-6 fold, 20–30 fold, and 600–800 fold above the therapeutic peak were tested, as neither OP nor its active metabolite, oseltamivir carboxylate (OC), could be detected at near-therapeutic concentrations. The transplacental transfer and accumulation of OC were assessed using the ** ^14^C antipyrine reference method. *Results*. OP was extensively metabolized to OC. In the 4 placentas with the highest concentration of OP, OC had a mean clearance index of 0.13 ± 0.08, suggesting that transplacental passage occurs at a relatively low rate. Measurable fetal accumulation occurred in the two placentas with the highest initial concentrations. *Conclusions*. Oseltamivir phosphate was extensively metabolized in the ex vivo model. Transplacental transfer of the metabolite was incomplete and accumulation was minimal.

## 1. INTRODUCTION

Oseltamivir phosphate (Tamiflu, Roche), (3R, 4R, 5S)-4-acetylamino-5-amino-3(1-ethylpropoxy)-1cyclohexene-1carboxylic acid, ethyl ester, phosphate (1 : 1) is an antiviral agent with activity against both influenza A and B [1]. Oseltamivir is one of two members of the class of antivirals known as neuraminidase inhibitors whose proposed mechanism of action is to interfere with the release of progeny viral particles from infected host cells, thereby preventing local spread of infection [2]. Both oseltamivir and zanamivir (Relenza, GlaxoSmithKline) are approved for the treatment of influenza A and B; however, only oseltamivir is administered orally and is approved for postexposure chemoprophylaxis [1].

Globally, influenza remains one of the most significant causes of febrile respiratory morbidity and mortality, as it affects approximately 20% of the world's population annually [2]. In the United States alone, epidemics of influenza are thought to be responsible for up to 36,000 deaths per year [3]. Immunoprophylaxis remains the cornerstone of controlling the spread of influenza, and annual vaccination is recommended for all persons who are at increased risk for complications from influenza infection [3]. Among those included within these high-risk groups, pregnant women are of particular interest because of the implications of maternal illness to her unborn child. The risks for severe illness and death related to influenza infection are known to be elevated in pregnant women, a concept historically supported by the disproportionate number of influenza-related maternal deaths during the pandemics of 1918 and 1957 [4]. Based on these risks, the CDC now recommends universal vaccination for pregnant women, regardless of gestational age. Despite these recommendations, however, the annual number of hospitalizations for seasonal influenza infection in pregnant women remains high [5, 6].

In addition to seasonal influenza, significant attention has recently been given to the particularly pathogenic strain of avian influenza, H5N1. A number of cases of human infection with H5N1 have been reported with a high case fatality rate, including a report of a pregnant woman who succumbed rapidly to viral pneumonia caused by H5N1 [7]. There is growing concern that H5N1 will cause the next influenza pandemic, yet no vaccine exits for this virulent strain of the virus [8].

There exists, therefore, great clinical interest in antiviral agents that may reduce the burden of annual clinical disease, and oseltamivir, in particular, may also have activity against avian influenza [9]. Unfortunately, limited data exist concerning the use of oseltamivir in pregnancy. The purpose of this study was to evaluate the metabolism and maternal-fetal transplacental passage of oseltamivir in the ex vivo human placental model.

## 2. MATERIALS ANDMETHODS

Term placentas (*n* = 6) were collected from either vaginal or otherwise uncomplicated cesarean deliveries in accordance with the University of Texas Southwestern Medical Center Institutional Review Board for Human Studies Guidelines, and they were transported to the laboratory in normal saline immediately after delivery. Both oseltamivir phosphate and its active metabolite oseltamivir carboxylate were obtained, with permission, from Roche (Basel, Switzerland).

The single cotyledon placental perfusion system was used as described by Schneider and Huch [10]. A fetal artery and vein on the chorionic plate were cannulated with 3.0 F and 5.0 F catheters, respectively. The fetal circulation was established by gently perfusing Eagle's minimal essential media including 3% bovine albumin and 0.5 units/mL of heparin (Sigma Chemical Co, St. Louis, Mo, USA). The cotyledon was initially examined for vascular integrity.

The isolated cotyledon and adjoining placental tissue were transferred to a temperature-controlled chamber (37°C) where the fetal circulation was perfused with media at 4.5–5.0 mL/min for 20 minutes to stabilize the pressure and determine if the cotyledon had a vascular leak. In general, we could not detect leaks of less than 2 cc/min; however, volumes into and out of the system were carefully measured, and when a change in volume was noted, the system was presumed to have a leak. The final pressure was generally about 35 mmHg. If the cotyledon failed to reach a stable baseline pressure or was found to have a leak, it was discarded. Finally, the maternal side of the catheterized cotyledon was sealed, and three 18-guage needles were inserted into the intervillous space of the selected cotyledon to reestablish the maternal circulation. Maternal flow rate was at 17 mL/min.

The maternal and fetal compartments each contained 150 cc of Eagle's minimal essential medium (pH 7.2–7.4), which was aerated with 95% oxygen and 5% carbon dioxide. Maternal-fetal transfer was performed with oseltamivir phosphate (OP) by adding the drug to the maternal circulation at concentrations that were approximately 5-6 fold (*n* = 2), 20–30 fold (*n* = 2), and 700–800 fold (*n* = 2) above the therapeutic peak. Supratherapeutic concentrations were studied, as neither OP nor its active metabolite, oseltamivir carboxylate (OC), could be detected when near-therapeutic concentrations were used.

In each of the 6 placentas that met study criteria, experiments were conducted for the first hour with both circulations open to determine the clearance index (CI) of the drug. During this portion of the experiment, 2–5 cc of outflow samples were collected from the venous sides of both the maternal and fetal circulations every ten minutes. Then, both systems were closed and recirculated for one additional hour to determine levels of accumulation. The collected fluid aliquots were then analyzed. The transport fraction of ^14^C antipyrine was used as a reference compound to determine the CI of oseltamivir carboxylate. A transport fraction of more than 30% for antipyrine was deemed representative of maternal-fetal circulatory match [10].

The high-pressure liquid chromatography (HPLC) assay of the prodrug oseltamivir phosphate is a modification of the procedure described by Sweeny et al. [11]. The HPLC analysis was carried out on Waters Associates instruments (Milford, Mass, USA) and consisted of a preparation of a standard curve to breach the range of oseltamivir used. All specimens and standards were extracted in aliquots of 0.5 mL. To each tube was added 0.5 mL of acetonitrile to precipitate blood and other proteins. The samples and standards were mixed and centrifuged at 800x gravity for 10 minutes, and the clear supernatant was injected into a Supelcosil LC-18-B Column (Supelco Chromatography Products, Bellefonte, Pa, USA). The HPLC conditions consisted of a mobile phase of 50% acetonitrile and 50% 0.01 M ammonium acetate. The sensitivity of the instrument was 0.005 AU at a wavelength of 230 nm. The flow rate of the mobile phase was 1.5 mL/min, and an injection volume of 40 microliters was used. The retention time of oseltamivir was 7.5 minutes. All concentrations of the drug were determined by measuring peak height of the extracted standards and perfusates. The HPLC method was validated; the reproducibility data are not shown.

The HPLC method for assaying the metabolite oseltamivir carboxylate was as follows. The drug was dissolved in water and diluted to anticipated therapeutic concentrations. The placental perfusion samples were aliquoted in 1.0 mL samples and made alkaline with 20 mL of 5N NaOH. The samples were then added to the C_18_ Sep-pack column (Waters Associate, Milford, Mass, USA) that had been activated by the following procedure: 1 mL of 0.1 M PO_4_ buffer (pH 6.1) was added to 0.1 gm/50 cc of 2-(N-morphalino) ethane sulfonic acid (MES), an ion buffer; this mixture was then washed through the column and the procedure repeated. One mL of methyl alcohol was added to the column and washed, followed by two washings of the phosphate buffer containing MES. After the sample had been pulled through by vacuum, the column was washed with 1 mL of PO_4_ buffer containing MES. At this time the active metabolite was washed off the Sep-pack column with phosphate buffer containing 5% acetonitrile. The HPLC assay parameter consisted of a 717 autosampler, a 486 detector, a 515 pump (Waters Associate instruments, Milford, Mass, USA), and a 0.1 millivolt Linear 1200 chart recorder (Scientific Marketing, Houston, Tex, USA). The HPLC column was a Supelcosil LC-18-DB, 25 cm × 4.6 mm, 5 *μ*m column (Supelco, Bellefonte, Pa, USA). Seventy-five microliters of extracted samples were injected into the system with a flow rate of 2.5 mL/min at detection settings of 0.01 AU, 220 nm wavelength with a metabolite retention time of about 6.4 minutes. The mobile phase was 5% acetonitrile in 0.1 M PO_4_ buffer (pH 6.1) containing 0.5 gm of MES per 500 mL. The standard curve was validated for within and between batch reproducibility and the lower limit of quantitation determined.

## 3. RESULTS

The recovery of OP and OC by HPLC analysis was greater than 80%. The minimum sensitivities for detection were 30 ng/mL and 2.4 ng/mL for OP and OC, respectively.

Previous pharmacokinetic studies of oseltamivir have determined that the peak plasma concentrations after a 75 mg twice-daily dosing regimen are 65.2 ng/mL for OP and 348 ng/mL for OC [1]. In our study, we were unable to detect either the prodrug or its active metabolite when levels in the perfusate were either in the therapeutic range or 5-6 fold above the therapeutic range. At higher concentrations (20- to 830-fold above the therapeutic range), we were able to detect OC in both the maternal and fetal circulation (Table 1). The results presented in the table represent the mean concentrations from the samples assayed in the open circulation. Mean concentrations were used to ensure that the values were representative of a steady state. The mean clearance index in these supratherapeutic concentrations was 0.13 ± 0.08. In a recirculated (i.e., closed-closed) system, OC accumulated in the fetal circulation only in the two placentas with the highest initial concentrations of OP; the mean accumulation in these cases was 17.0 ± 14.6 ng.

## 4. DISCUSSION

The pharmacokinetics of oseltamivir have been studied in geriatric and pediatric populations, and treatment trials have demonstrated the safety and efficacy of the drug in these populations, both of which are considered high-risk for complicated influenza-related illness [2]. Little is known, however, regarding the pharmacokinetics or safety of oseltamivir in pregnancy. Oseltamivir is classified as Pregnancy Category C, as animal studies have suggested that an adverse effect on the fetus may exist, but there have been no well-controlled studies conducted in humans. Animal studies [12] for effects on embryo-fetal development have been conducted with rats and rabbits who were administered oseltamivir by the oral route. Relative doses were up to 100 times the human exposure in the rat and up to 50 times in the rabbit. Fetal exposure was seen in both species, and there was a dose-dependant increase in the incidence of minor skeletal deformities. The individual incidences of each anomaly, however, remained within the background rates of occurrence of the species studied. Although we are unable to examine the fetal effects of exposure to oseltamivir in the ex vivo model, we are not aware of any other study evaluating the metabolism and transplacental passage of oseltamivir in the human placenta.

Oseltamivir phosphate is administered orally as an ethyl ester prodrug which is extensively converted by hepatic esterases to the active compound oseltamivir carboxylate [1]. Approximately 80% of an orally administered dose reaches the circulation as OC, and the metabolite has excellent penetration into tissues such as the lung, nasal mucosa, and middle ear [13]. The peak plasma concentrations are lower and the half-life is shorter for OP than for OC, respectively, so the placenta of a pregnant woman would presumably be exposed to higher sustained levels of OC than the prodrug. Since, however, no pharmacokinetic studies have been conducted in pregnant subjects, we chose to examine the prodrug in our placental perfusion model. Subsequently measuring the concentrations of the metabolite in the maternal and fetal circulations enabled us to estimate both the extent to which the drug was metabolized and the efficiency with which the metabolite transfers from one compartment to another. Oseltamivir carboxylate does not undergo further metabolism in vivo and is excreted unchanged in urine [13]; therefore, the clearance index obtained from our model is likely a valid estimate of maternal-fetal transfer.

Our results suggest that OP is extensively metabolized to OC in the ex vivo human placental model. Although hepatic esterases are primarily responsible for the hydrolysis of OP to OC in vivo, serum esterases can also effect significant conversion of the prodrug to the metabolite. Lindegardh et al. [14] examined the ex vivo metabolism of oseltamivir and found that up to 31.8% of the prodrug was converted into the metabolite after 4 hours of exposure to human serum. This conversion was not completely arrested even when the samples were placed on ice, but it was inhibited by the addition of the esterase inhibitor dichlorvos. In our model, most residual fetal blood is removed by perfusing the fetal artery and vein after initial cannulation; however, the “flushing” of the intervillous space is less complete in the maternal circulation, and it is possible that the perfusate was exposed to residual maternal serum and the proteins contained therein. The placenta itself, however, also contains esterase activity. Histochemical analysis of the human placenta has demonstrated a high concentration of “nonspecific” esterase in the trophoblast of the villi and a variable concentration in the villous cores [15]. We are unable to determine in our study whether OP was metabolized to OC by serum esterases, placental esterases or both, but our results suggest that this conversion was extensive.

The transplacental transfer of a substance is regulated by a number of variables, including molecular weight, ionic charge, concentration in the maternal plasma, maternal blood flow rate, protein binding, and placental metabolism [16]. Most compounds with a molecular weight less than 500 daltons diffuse readily through the placenta [16]. Given that oseltamivir carboxylate has a molecular weight of 312.4 daltons and has low protein binding (3%), [1] it was somewhat surprising that the transplacental passage of this compound was incomplete in the ex vivo model. Although OC is not thought to be further metabolized, another metabolite of OP has been identified by Sweeny et al. [11]. They described a novel metabolite which was recovered from the urine, plasma, and tissue samples of rats who were administered OP by the oral route. The (R)-*ω*-carboxyilic acid metabolite was identified by HPLC and NMR analysis; and it was the second most abundant metabolite in these specimens after the active neuraminidase inhibitor. In the present study, we did not evaluate for the presence of *ω*-hydroxylated products, and if placental metabolism of OP yielded these metabolites, it is possible—depending on the distribution of the products of hydrolysis—that our estimated clearance index of OC is falsely low. Importantly, however, very high concentrations of OP had to be studied before either the prodrug or the active metabolite could be detected, and accumulation in the fetal compartment was minimal.

Oseltamivir is the only orally bioavailable antiviral medication that is recommended for chemoprophylaxis and treatment of influenza in the United States, and the demand for this drug is likely to increase. The adamantane class of antivirals, including amantadine and rimantadine, has activity against influenza A, but sufficient resistance has developed in contemporary strains of influenza that the CDC has recommended avoiding the use of these agents until susceptibility can be reestablished [3]. Resistance to oseltamivir, conversely, has been observed infrequently, and the resistant strains identified in clinical samples appear to have reduced infectivity and potential for transmission [9]. Oseltamivir has also demonstrated efficacy—both in vitro and in animal models—against the H5N1 and H9N2 strains of avian influenza for which no effective vaccine exists [9]. To date, the World Health Organization has reported 329 confirmed cases of avian influenza A/(H5N1) with 201 deaths (61% case-fatality rate), and concern that a mutated H5N1 could result in the next pandemic has caused a number of nations and international agencies to generate a stockpile of oseltamivir [17]. The potential global importance of this medication is quite evident, but it is unfortunate that such a paucity of data exists concerning its use in pregnant women, especially since they are more likely to develop serious complications from influenza infection than their nonpregnant counterparts.

## 5. CONCLUSIONS

Our study suggests that oseltamivir phosphate is extensively metabolized in the ex vivo human placental model. The active metabolite oseltamivir carboxylate was identified in the maternal and fetal circulations when very high doses of the prodrug were studied, and the transplacental transfer of the metabolite was incomplete in our model. Additional studies are needed to better characterize the pharmacokinetic behavior of oseltamivir in pregnancy to assess the need for dosing adjustments and the effects of the medication on the developing fetus.

## Figures and Tables

**Table 1:** Mean maternal and fetal concentrations of oseltamivir phosphate and oseltamivir carboxylate from 6 term placental cotyledons perfused with supratherapeutic concentrations of oseltamivir phosphate.

	Maternal (ng/ml)		Fetal (ng/ml)	
Placenta	OP*	OC	OP	OC
1	320 (5x)	ND	ND	ND
2	400 (6x)	ND	ND	ND
3	1 300 (20x)	86	ND	4.3
4	2 160 (30x)	140	30	5
5	51 000 (780x)	90	7300	9
6	54 000 (830x)	96	3300	2.4

*concentration relative to therapeutic dose in parentheses. ND = nondetectable.
